# ApoE and apoC-III-defined HDL subtypes: a descriptive study of their lecithin cholesterol acyl transferase and cholesteryl ester transfer protein content and activity

**DOI:** 10.1186/s12944-020-01291-x

**Published:** 2020-05-25

**Authors:** Mateo Amaya-Montoya, Jairo A. Pinzón-Cortés, Lina S. Silva-Bermúdez, Daniel Ruiz-Manco, Maria C. Pérez-Matos, Mario A. Jiménez-Mora, Carlos O. Mendivil

**Affiliations:** 1 Universidad de los Andes Medical School, Carrera 7 # 116-05, Of. 413, Bogotá, Colombia; 2 Department of Internal Medicine, Fundación Santa Fe de Bogotá, Section of Endocrinology, Carrera 7 No. 117 – 15, Bogotá, Colombia

**Keywords:** HDL, Lecithin cholesterol acyltransferase, Cholesterol ester transfer protein, Apolipoprotein E, Apolipoprotein C-III, Reverse cholesterol transport

## Abstract

**Background:**

The functionality of high-density lipoproteins (HDL) is a better cardiovascular risk predictor than HDL concentrations. One of the key elements of HDL functionality is its apolipoprotein composition. Lecithin-cholesterol acyl transferase (LCAT) and cholesterol-ester transfer protein (CETP) are enzymes involved in HDL-mediated reverse cholesterol transport. This study assessed the concentration and activity of LCAT and CETP in HDL subspecies defined by their content of apolipoproteins E (apoE) and C-III (apoC-III) in humans.

**Methods:**

Eighteen adults (ten women and eight men, mean age 55.6, BMI 26.9 Kg/m^2^, HbA1c 5.4%) were studied. HDL from each participant were isolated and divided into four subspecies containing respectively: No apoE and no apoC-III (E-C-), apoE but not apoC-III (E + C-), apoC-III but no apoE (E-C+) and both apoE and apoC-III (E + C+). The concentration and enzymatic activity of LCAT and CETP were measured within each HDL subspecies using immunoenzymatic and fluorometric methods. Additionally, the size distribution of HDL in each apolipoprotein-defined fraction was determined using non-denaturing electrophoresis and anti-apoA-I western blotting.

**Results:**

HDL without apoE or apoC-III was the predominant HDL subtype. The size distribution of HDL was very similar in all the four apolipoprotein-defined subtypes. LCAT was most abundant in E-C- HDL (3.58 mg/mL, 59.6% of plasma LCAT mass), while HDL with apoE or apoC-III had much less LCAT (19.8, 12.2 and 8.37% of plasma LCAT respectively for E + C-, E-C+ and E + C+). LCAT mass was lower in E + C- HDL relative to E-C- HDL, but LCAT activity was similar in both fractions, signaling a greater activity-to-mass ratio associated with the presence of apoE. Both CETP mass and CETP activity showed only slight variations across HDL subspecies. There was an inverse correlation between plasma LCAT activity and concentrations of both E-C+ pre-beta HDL (*r* = − 0.55, *P* = 0.017) and E-C- alpha 1 HDL (*r* = − 0.49, *P* = 0.041). Conversely, there was a direct correlation between plasma CETP activity and concentrations of E-C+ alpha 1 HDL (*r* = 0.52, *P* = 0.025).

**Conclusions:**

The presence of apoE in small HDL is correlated with increased LCAT activity and esterification of plasma cholesterol. These results favor an interpretation that LCAT and apoE interact to enhance anti-atherogenic pathways of HDL.

## Background

Evidence from multiple observational studies has demonstrated a negative association between plasma concentrations of high-density lipoprotein cholesterol (HDL-C) and the risk of cardiovascular disease (CVD) [1–4]. However, medications aimed at raising HDL-C have failed to reduce the incidence of CVD in clinical trials. Several high-density lipoprotein (HDL)-raising agents have failed to prevent CVD including cholesteryl ester transfer protein (CETP) inhibitors, fibrates and niacin [5–8]. This apparent paradox can be explained by the fact that HDL functionality, rather than HDL-C concentration, is the relevant measure associated with CVD prevention [9, 10]. HDL functionality is a broad concept that includes reverse cholesterol transport (RCT), induction of nitric oxide synthesis, reduction in the expression of cell adhesion molecules and antioxidant activity [11]. Lecithin-cholesterol acyl transferase (LCAT) and CETP are enzymes strongly involved in HDL metabolism and functionality. LCAT transfers an acyl group from lecithin to free cholesterol, forming cholesterol esters that move to the core of the HDL particle and are later taken up by the liver [12, 13]. Meanwhile, CETP catalyzes the transfer of cholesterol esters from HDL to apoB lipoproteins in exchange for triglycerides [14]. Subsequently, these apoB lipoproteins are also removed from circulation by the liver, completing an indirect pathway of RCT [15].

The concentration and activity of LCAT and CETP are modulated by different factors. For example, polyunsaturated fatty acids (PUFA) reduce expression of the LCAT gene and synthesis of LCAT in vitro [16]. Sphingomyelin [17], oxidized lipids [18, 19], n− 3 fatty acids [20] and trans-unsaturated fatty acids [21] inhibit LCAT in vitro. Meanwhile, a high dietary intake of cholesterol or omega-3 PUFA and the use of fibrates upregulate expression of the CETP gene [22, 23]. Plasma CETP activity and mass are also increased in parallel with higher plasma concentrations of bile acids [24], and decreased in patients with hypothyroidism [25]. Nonetheless, the modulation of human LCAT and CETP by components of HDL in vivo is insufficiently understood.

All lipoproteins harbor a repertoire of small apolipoproteins, which act as modulators of their metabolic fate. Two of these small apolipoproteins are apoE and apoC-III. ApoE mediates the clearance of very low-density lipoproteins (VLDL), intermediate-density lipoproteins (IDL) and chylomicron remnants via the low-density lipoprotein (LDL) receptor, LDL receptor-related protein-1 (LRP-1) or heparan sulfate proteoglycans [26, 27]. Contrastingly, apoC-III strongly inhibits the clearance of all apoB-lipoproteins [28, 29]. ApoC-III also impairs the catabolism of triglyceride-rich lipoproteins and stimulates hepatic VLDL assembly and secretion [28, 30]. However, the role of apoE and apoC-III on HDL physiology is much less understood. It is known that besides apoA-I (the natural cofactor of LCAT) [31], apoE is able to activate LCAT in vitro [32], while an increase in the apoC-III content of synthetic HDL exerts the opposite effect [33]. ApoE is able to partially rescue LCAT activity In apoA-I knockout mice, albeit only in VLDL and LDL, not in HDL [34]. Despite their major relevance in lipoprotein metabolism, the influence of apoE and apoC-III over LCAT and CETP in humans is very poorly understood.

With this background, this study analyzed the distribution of LCAT and CETP mass and activity in multiple HDL subclasses defined by their content of apoE and apoC-III and molecular size, in normal weight and overweight adult humans.

## Methods

### Study design and participants

The study included 18 participants between 33 and 76 years old, selected from a project about new biomarkers of insulin resistance [34]. Exclusion criteria were known diabetes mellitus or use of anti-diabetic medications, other endocrine disorders, diseases of the exocrine pancreas, pregnancy or use of oral anticoagulants or lipid-lowering drugs. Individuals who had symptoms of an acute viral or bacterial infection, or with plasma high-sensitivity C-reactive protein (hsCRP) concentrations higher than 10 mg/L, were also excluded. Blood samples were drawn in EDTA (ethylenediaminetetraacetic acid) tubes after an 8-h fast. Plasma was promptly separated, supplemented with a preserving cocktail (benzamidine, phenylmethylsulphonyl fluoride and gentamicin), aliquoted and stored at − 80 °C for later analyses. Plasma concentrations of apoA-I were measured using a nephelometric method [35] and fasting plasma glucose (FPG), plasma lipids and creatinine using conventional colorimetric assays (Biosystems, Barcelona, Spain). Glycated hemoglobin A1c (HbA1c) was determined using a National Glycohemoglobin Standardization Program-certified boronate affinity technique (NycoCard™ Reader II, Alere Technologies, Oslo, Norway).

### HDL isolation and separation

Plasma was passed through Acrodisc® 5 μm filters (Pall Corporation, Port Washington, NY, USA), in order to remove fibrin impurities. Then, HDL was purified from plasma using immunoaffinity chromatography as follows: Sepharose 4B™ resin bound to goat polyclonal anti-human apoA-I antibodies (Academy Bio-Medical, Houston, Texas, USA) was loaded into 10 mL Poly-Prep® chromatography columns (Bio-Rad, Hercules, California, USA), and 1 mL of plasma was incubated overnight in the column. The unbound fraction was collected by gravity flow and stored at − 80 °C. The bound fraction was eluted using 3 consecutive washes of 3 M sodium thiocyanate (NaSCN) and one last phosphate-buffered saline (PBS) wash followed by concentration and desalting in 10 KDa molecular weight cutoff Amicon® filters (Merck Millipore, Billerica, MA, USA) until 1 mL of fraction was achieved. Then two PBS washes were performed to remove any NaSCN left over in the column before another use. The same process was carried out with columns containing goat polyclonal anti-human apoC-III and anti-human apo-E antibodies, in that order. At the end, total HDL from each participant was divided in 4 subfractions: HDL without apoE or apoC-III (E-C-), HDL with apoE but without apoC-III (E + C-), HDL without apoE but with apoC-III (E-C+) and HDL with both apoE and apoC-III (E + C+). The efficiency of the immunoaffinity columns was 95–98% for all study subjects. The final buffer for all HDL subfractions was PBS.

### Determination of enzymatic content and activity in HDL subfractions

LCAT concentration was determined using an immunoenzymatic, double-sandwich assay (ALPCO Diagnostics, Salem, NH, USA), in which capture is performed by a first monoclonal antibody against LCAT (MoAb 36,486) and detection is performed with a different, horseradish peroxidase (HRP)-labeled anti-LCAT monoclonal antibody (MoAb 36,487). After incubation with a substrate solution and termination with a stop reagent, the intensity of absorbance at 492 nm was read in a Synergy HT microplate reader using Gen5 software (BioTek, Winooski, VT, USA). CETP concentration was determined using an immunoenzymatic sandwich assay (ALPCO Diagnostics, Salem, NH, USA), with MoAb 3-11D as capture antibody and HRP-labeled MoAb 14-8F as detection antibody.

Measurement of LCAT activity was done using a fluorometric assay (Calbiochem, Darmstadt, Germany). The assay is based on the incubation of a substrate that fluoresces at 470 nm with the study samples. After LCAT in the samples removes a fatty acid from the substrate and transfers it to cholesterol, the substrate loses fluorescence at 470 nm and gains fluorescence at 390 nm. Thus, LCAT activity was measured as change in 470/390 emission intensity by comparison against a calibration curve based on dilutions of a plasma pool. CETP was also measured with a fluorometric assay (Abcam, Cambridge, UK). The assay principle involves incubating the sample with a mixture that contains a self-quenched fluorescent neutral lipid and an acceptor molecule. Action of CETP in the sample results in transfer of the neutral lipid to the acceptor molecule and an increase in its fluorescence (excitation at 465 nm, emission at 535 nm). For CETP too, activity was measured by comparison against a calibration curve based on dilutions of a plasma pool. Enzymatic activity was expressed as percent of activity in the plasma pool, and was therefore expressed in Arbitrary Units (AU). All measurements were performed in duplicate.

The cholesteryl ester content of plasma and HDL subfractions was measured using the Abcam cholesteryl ester kit (ab65359, Abcam, Cambridge, UK). The assay is based on two simultaneous reactions for cholesterol determination, one of which includes cholesterol esterase as part of the reaction mixture, while the other does not. Cholesteryl esters are then calculated by subtraction as (total cholesterol - free cholesterol). Phosphatidylcholine in plasma and HDL subfractions was measured using the Abcam fluorometric assay (ab83377, Abcam, Cambridge, UK). The test is based on an enzyme-coupled reaction that hydrolyzes phosphatidylcholine and releases choline, which in turn reacts with the OxiRed probe and generates fluorescence at 587 nm wavelength.

### Determination of HDL size distribution

HDL contained in each of the four apoC-III and apoE-defined fractions were separated by size using non-denaturing polyacrylamide gradient gel electrophoresis (NDPAGGE). Twenty-five microliters of each immunofraction plus 25 μl of sample buffer were loaded into a 4–30% polyacrylamide gradient gel (Jule Inc., Milford, CT, USA). Wells 1 and 10 were loaded with molecular size standards (Amersham HMW Native Marker Kit, GE Healthcare, Little Chalfont, UK) and gels were run for 24 h at constant 70 V. Then, contents of the gel were transferred to a 0.45 μm pore size polyvinylidene fluoride (PVDF) membrane (Pall Corporation, New York, NY, USA) in a wet transfer apparatus at 30 V for 24 h. The lanes containing the MW markers were cut from the rest of the membrane, stained in 0.2% amido black solution for 20 min and stored for later photographing. The rest of the membrane was blocked with 5% powder low-fat milk, incubated with an HRP-conjugated goat anti-human apoA-I antibody (Academy Bio-medical, Houston, TX, USA), and revealed using 3,3′,5,5′-tetramethylbenzidine as substrate. Later, the marker lanes and the rest of the membrane were placed side to side and photographed in a Bio-Rad ChemiDoc™ MP gel documenter. Using the molecular size standards as reference, the intensity of the bands/smears in each size range fractions was quantitated in Image Lab™ software. Size fractions were defined as follows: prebeta HDL: < 7.1 nm, alpha 3 HDL: 7.1–8.2 nm, alpha 2 HDL: 8.2–9.5 nm and alpha 1 HDL: 9.5–12.2 nm. The concentration of apoA-I in each HDL size subfraction was estimated by multiplying the proportion of apoA-I within that fraction by the directly measured total plasma apoA-I. Laboratory procedures were executed at the Diabetes, Lipids and Metabolism laboratory of Universidad de Los Andes, following current institutional biosafety protocols.

### Statistical analyses

A sample size of eighteen participants provided 87% power to detect a true difference of at least 8% in LCAT activity between two HDL subtypes, assuming a 5% variation coefficient in LCAT activity [18], at a 5% significance level. The distribution of plasma apoA-I across HDL subfractions was compared in a 2-way analysis of variance (ANOVA) model in which apoA-I concentration in each subfraction was the dependent variable, and HDL size and immunofraction were fixed factors. Enzyme concentrations and activities were compared among HDL subtypes using a 1-way ANOVA in which HDL immunofraction was the only fixed factor. When global ANOVA was significant, post-hoc comparisons against the reference fraction (E-C-) were done using Scheffé’s method. Comparisons of numeric variables between groups of participants were performed using Mann-Whitney’s U test. The significance of differences in proportions between groups of participants was assessed using Fisher’s exact test. All tests were done at a 0.05 significance level and all reported *p*-values are 2-sided.

## Results

The study included eight male and ten female participants, with mean age 55.6 +/− 11.2 years and mean body-mass index (BMI) 26.9 +/− 4.0 kg/m^2^. On average, women had higher plasma total cholesterol, HDLc and LDLc. Women also had significantly higher plasma apoA-I levels (121.4 +/− 14.3 mg/dL versus 97.7 +/− 23.0 mg/dL in men, *P* = 0.016). Plasma LCAT concentration was 6.3 +/− 2.2 mg/dL in women and 5.7 +/− 2.2 mg/dL in men, while plasma CETP concentrations were 1.9 +/− 0.7 mg/dL in women and 1.7 +/− 0.7 mg/dL in men. The relative activity of the enzymes under study was not different between sexes: 98.7 +/− 13.7 AU in women versus 101.7 +/− 6.1 AU in men for CETP and 96.1 +/− 22.3 AU in women versus 96.8 +/− 18.4 AU in men for LCAT (Table 1).

**Table 1:** Characteristics of study participants. eGFR: Estimated glomerular filtration rate, CETP: Cholesteryl ester transfer protein, LCAT: Lecithin cholesterol acyl transferase. Data are mean +/− SD unless stated otherwise

Sex (F:M)	10:8
Age (years)	55.6 +/− 11.2
Weight (kg)	72.0 +/− 12.9
Height (m)	163.5 +/− 9.2
Body-mass index (kg/m^2^)	26.9 +/− 4.0
Percent body fat (%)	33.1 +/− 7.1
Percent abdominal fat (%)	9.4 +/− 3.9
Percent lean mass (%)	63.6 +/− 6.7
Systolic blood pressure (mmHg)	117.8 +/− 14.6
Diastolic blood pressure (mmHg)	73.8 +/− 11
Fasting plasma glucose (mg/dl)	96.1 +/− 10.6
Glycated hemoglobin (HbA1c) (%)	5.4 +/− 1.0
eGFR (ml/min)	93.2 +/− 18.0
Total cholesterol (mg/dl)	195.5 +/− 52.4
Triglycerides (mg/dl)	154.2 +/− 58.4
HDL cholesterol (mg/dl)	43.8 +/− 15.8
LDL cholesterol (mg/dl)	126.3 +/− 44.4
C-reactive protein (mg/l)	1.9 +/− 2.6
Plasma apoA-I (mg/dl)	110.9 +/− 21.8
Plasma CETP (microg/ml)	1.8 +/− 0.7
Plasma LCAT (microg/ml)	6.0 +/− 2.1
Plasma CETP activity (AU)	100.0 +/− 10.8
Plasma LCAT activity (AU)	96.4 +/− 20.1

### HDL with apoE or apoC-III are minority

**Fig. 1:**
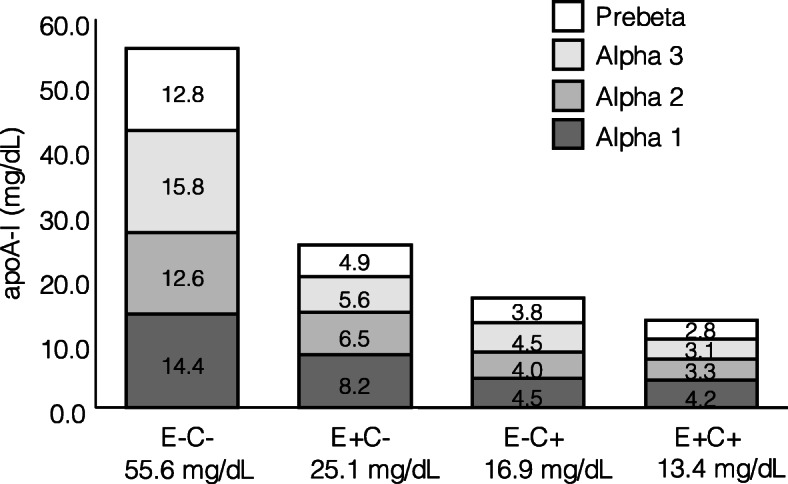
Distribution of plasma apoA-I across HDL subfractions defined by size, apoE and apoC-III content. HDL size ranges are as follows: Prebeta: < 7.1 nm, alpha 3: 7.1–8.2 nm, alpha 2: 8.2–9.5 nm, alpha 1: 9.5–12.2 nm (*n* = 18, 10 women and 8 men)

The predominant subfraction of HDL (as reflected by its concentration of apoA-I) was E-C- in most study participants. On average, E-C- HDL represented 50.1% of plasma apoA-I, followed by E + C- (22.6%), E-C+ (15.2%) and E + C+ (12.1%) (*p* < 0.001 for difference across subfractions) (Fig. 1). There was significantly more apoA-I in E-C- than in E + C-, E-C+ or E + C+ HDL (*p* < 0.001 for each of the three pairwise comparisons). Overall, 34.7% of HDL contained any apoE while 27.3% contained any apoC-III.

### LCAT concentration and activity vary across HDL subfractions

**Fig. 2:**
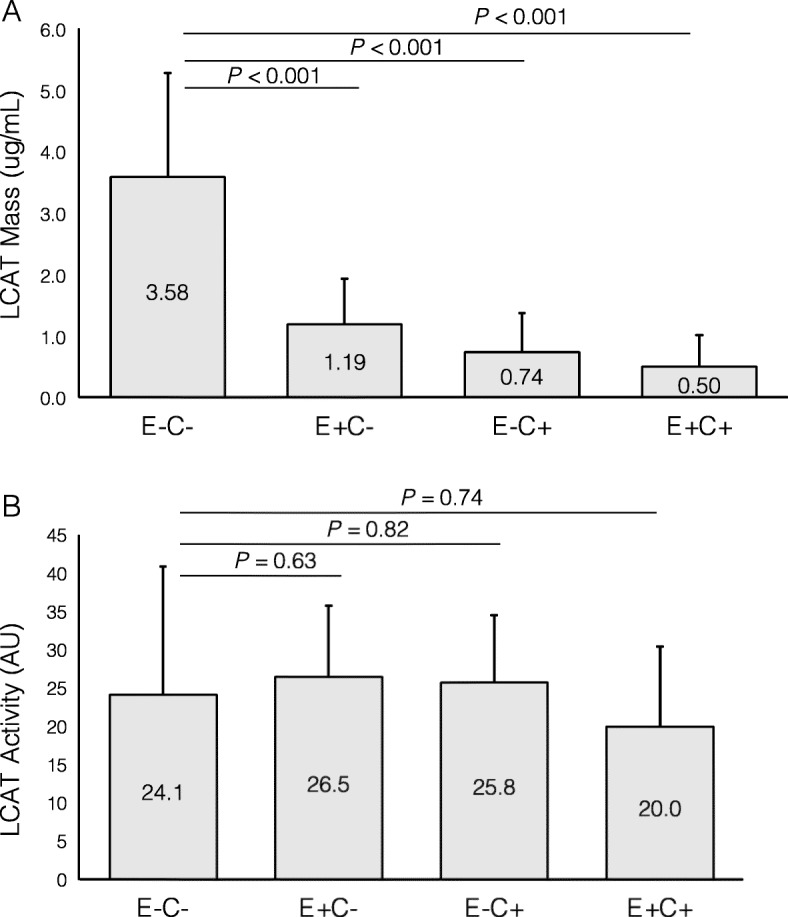
Lecithin cholesterol acyl transferase (LCAT) concentration and activity in HDL subfractions defined by their apoE and apoC-III content. **a** LCAT concentration. **b** LCAT activity (*n* = 18, 10 women and 8 men)

Most plasma LCAT mass (3.58 microg/mL, 59.6%) was concentrated in the E-C- HDL subfraction. The remaining fractions with either apoE or apoC-III had similarly lower LCAT concentrations: 1.19 microg/mL in E + C-, 0.74 microg/mL in E-C+ and 0.50 microg/mL in E + C+ (*p* < 0.001 for the difference across groups). The difference in LCAT concentration between E-C- and every other HDL subfraction was statistically significant (*p* < 0.001 for each of the three pairwise comparisons) (Fig. 2, upper panel). Surprisingly, this heterogeneity in LCAT protein distribution was not paralleled by LCAT activity, which had a different distribution. The E-C- fraction had a relative LCAT activity of 24.1 AU (25% of total plasma LCAT activity), and the highest LCAT activity was found in the in E + C- fraction (26.5 AU, 27.4% of total plasma LCAT activity) (*P* = 0.35 for difference across groups). HDL in the E-C+ and E + C+ fractions contained respectively 25.8 and 20.0 AU of LCAT activity (Fig. 2, lower panel). The ratio of LCAT activity / LCAT mass was 27.5 AU*mL/microg in HDL containing apoE (E + C- plus E + C+), and 11.6 AU*mL/microg in HDL not containing apoE (E-C- plus E-C+).

### CETP concentration and activity are similar across HDL subfractions

**Fig. 3:**
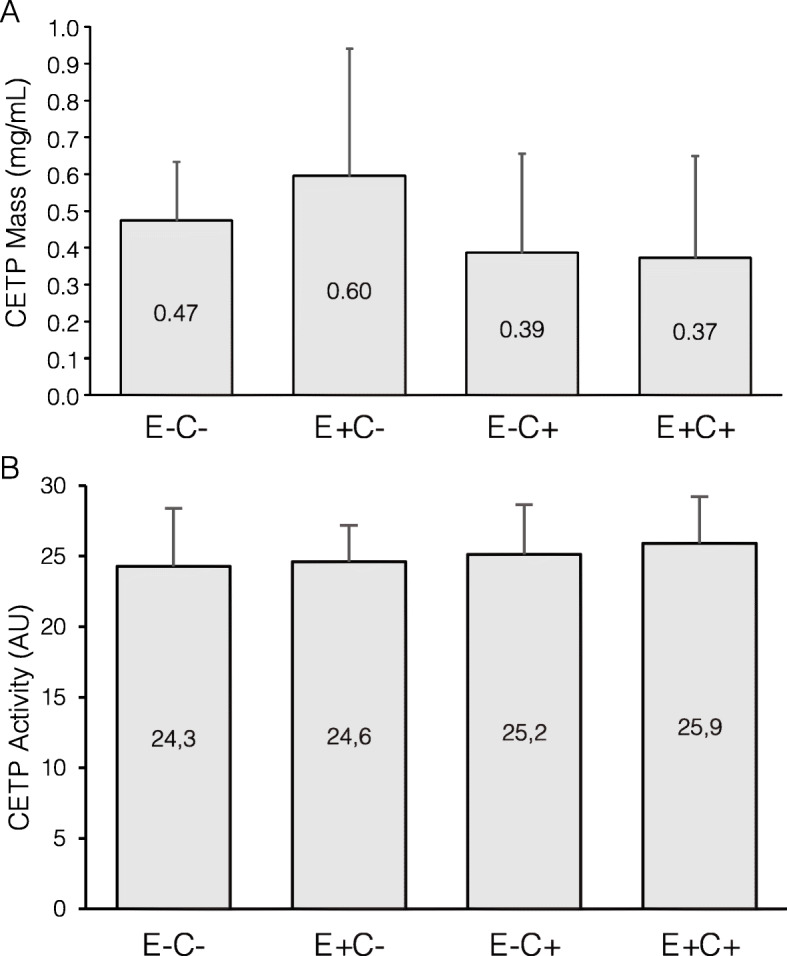
Cholesteryl ester transfer protein (CETP) concentration and activity in HDL subfractions defined by their apoE and apoC-III content. **a** CETP concentration. There was no significant difference across subfractions (overall ANOVA *P* = 0.48). **b** CETP activity. There was no significant difference across subfractions (overall ANOVA *P* = 0.50) (*n* = 18, 10 women and 8 men)

Most CETP was concentrated in the E + C- subfraction (0.60 mg/mL, 32.5% of total plasma CETP), while the other three types of HDL contained respectively 25.9% (E-C-), 21.1% (E-C+) and 20.4% (E + C+) of plasma CETP (*P* = 0.49 for comparison across fractions) (Fig. 3, upper panel). CETP activity showed a very homogeneous distribution across HDL subtypes (*P* = 0.50, Fig. 3, lower panel).

### Lipid composition of HDL subtypes

ApoC-III containing HDL displayed a higher triglyceride-to-apoA1 molar ratio, while E-C- HDL were poorer in triglycerides relative to the other fractions (*P* = 0.01 for global ANOVA, Table 2). Despite numerically higher phosphatidylcholine-to-apoA1 molar ratios in the apoE or apoC-III containing HDL, this difference did not achieve statistical significance. The cholesterol and cholesteryl ester contents of the four HDL subtypes were numerically similar, and not statistically different.

**Table 2:** Molar ratios of lipids to apoA-I in the four HDL subfractions. Data in the first row are mean lipid to apoA-I molar ratios +/− SD. The second row of each analyte shows the significance values from a post-hoc pairwise comparison versus the E-CIII- fraction. The third row of each analyte shows the proportion of each lipid in that fraction as a percentage of the total mass of such lipid in HDL. The last row shows the significance value from a global one-way ANOVA for the analysis of lipid to apoA-I molar ratios by subfractions

		E-CIII-	E + CIII-	E-CIII+	E + CIII+
**Triglycerides**	to apoA-I molar ratio	0.47 +/− 0.22	1.09 +/− 1.41	2.73 +/− 3.48	2.41 +/− 1.87
	*p*-value versus E-CIII-	–	0.88	0.035	0.09
	% of total mass in HDL	25.2%	17.2%	29.8%	27.7%
	*p*-value from ANOVA	0.01			
**Cholesterol**	to apoA-I molar ratio	26.6 +/− 18.7	51.5 +/− 68.4	48.1 +/− 32	52.6 +/− 38.1
	*p*-value versus E-CIII-	–	0.43	0.55	0.39
	% of total mass in HDL	40.7%	22.5%	20.8%	15.8%
	*p*-value from ANOVA	0.26			
**Phosphatidylcholine**	to apoA-I molar ratio	109.2 +/− 69.3	317.6 +/− 367.7	455.7 +/− 547.3	488.2 +/− 669.2
	*p*-value versus E-CIII-	–	0.65	0.21	0.15
	% of total mass in HDL	27.1%	27.0%	26.0%	19.7%
	*p*-value from ANOVA	0.088			
**Cholesterol esters**	to apoA-I molar ratio	14.6 +/− 16	23 +/− 29.9	16.3 +/− 37.9	30 +/− 103
	*p*-value versus E-CIII-	–	0.98	0.99	0.89
	% of total mass in HDL	48.6%	28.8%	12.3%	10.1%
	*p*-value from ANOVA	0.86			

### The concentration of some apolipoprotein and size-defined HDL correlates with plasma LCAT and CETP activity

The concentration of apoA-I in the E-C+ pre-beta HDL subfraction correlated inversely with total plasma LCAT activity (*r* = − 0.55, *P* = 0.017, Table 3). Similarly, apoA-I concentrations in the E-C- alpha 1 HDL subfraction correlated inversely with total plasma LCAT activity (r = − 0.49, *P* = 0.041). On the other hand, concentrations of larger E-C+ HDL (alpha 1) exhibited a positive correlation with plasma CETP activity (*r* = 0.52, *P* = 0.025). For the E-C+ HDL subtype, the correlation between HDL concentration and CETP activity was not significant for prebeta HDL, but tended to increase for HDL of larger sizes.

**Table 3:** Correlations between apoA-I mass within HDL size-defined subfractions and plasma enzymatic activities. Data are Spearman linear correlation coefficients. Asterisks denote correlations significantly different from zero

Correlations with LCAT activity	**E-CIII-**	**E + CIII-**	**E-CIII+**	**E + CIII+**
Alpha 1 HDL	−0.49*	−0.30	−0.35	−0.10
Alpha 2 HDL	−0.01	−0.29	−0.37	0.02
Alpha 3 HDL	0.33	−0.13	− 0.30	0.04
Pre-Beta HDL	0.24	−0.08	−0.55*	0.06
Correlations with CETP activity	**E-CIII-**	**E + CIII-**	**E-CIII+**	**E + CIII+**
Alpha 1 HDL	0.16	−0.10	0.52*	0.46
Alpha 2 HDL	0.04	−0.07	0.39	0.34
Alpha 3 HDL	−0.03	0.11	0.28	0.28
Pre-Beta HDL	0.11	−0.01	0.13	0.05

### The distribution of LCAT and CETP concentration and activity was not influenced by BMI

**Fig. 4:**
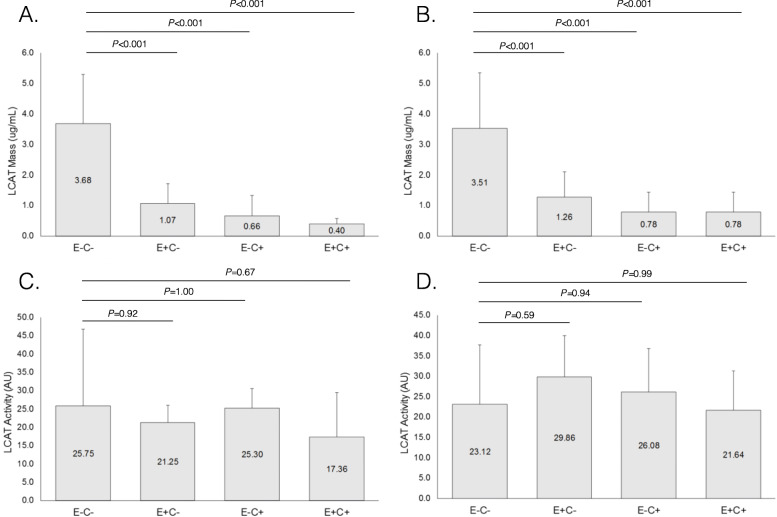
Lecithin cholesterol acyl transferase (LCAT) concentration and activity in HDL subfractions defined by their apoE and apoC-III content, in patients with body-mass index below 25 Kg/m^2^ (*n* = 7, left side, (**a** and **c**) or equal to or greater than 25 Kg/m^2^ (*n* = 11, right side, (**b** and **d**)

**Fig. 5:**
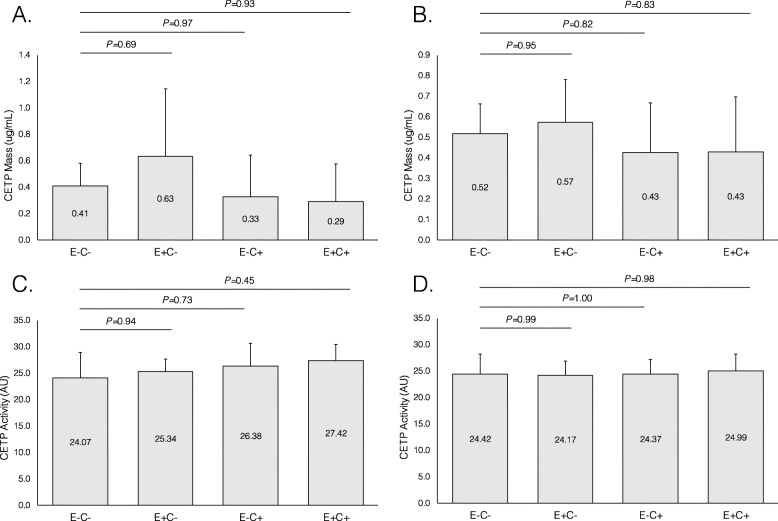
Cholesteryl ester transfer protein (CETP) concentration and activity in HDL subfractions defined by their apoE and apoC-III content, in patients with body-mass index below 25 Kg/m^2^ (*n* = 7, left side, (**a** and **c**) or equal to or greater than 25 Kg/m^2^ (*n* = 11, right side, (**b** and **d**)

As a sensitivity analysis, the findings concerning enzymatic concentrations and activities were compared between participants with a normal body-mass index (BMI<25), and participants with excess body weight (overweight or obesity, BMI> = 25). Just like in the complete study sample, in each of the two subgroups the concentration of LCAT was significantly higher in E-C- HDL, but enzymatic activity was similarly distributed across the four HDL subtypes (Fig. 4). Also in each of the two BMI-defined subgroups, CETP concentration and activity were very similar among the four HDL subtypes (Fig. 5).

## Discussion

The results of this in vivo study reveal the association between small apolipoproteins and the activity of enzymes involved in HDL-mediated reverse cholesterol transport. HDL with apoE have a greater ratio of LCAT activity to mass, signaling a greater degree of LCAT activation. Contrastingly, CETP mass and activity were not associated with the apoE or apoC-III composition of HDL. As reported previously, apoE and/or apoC-III-containing HDL were a minority of total plasma HDL [36], yet they are different from E-C- HDL in their enzymatic activity. These findings suggest an interaction between LCAT and apoE in HDL that favors cholesterol esterification, a critical step for its transport to the liver.

LCAT was most abundant in E-C- HDL, while HDL with either apoE or apoC-III contained similar amounts of LCAT. Despite having lower LCAT masses compared to E-C-, HDL with apoE or apoC-III had very similar LCAT activities, suggesting that the presence of either small apolipoprotein, or a factor closely associated with them, influences LCAT. Of note, the simultaneous presence of apoE and apoC-III in HDL was not accompanied by higher LCAT activity. The essential role of apoA-I as an LCAT cofactor is well documented, but very little is known about the impact of other small apolipoproteins on the activity of this crucial enzyme. Studies in apoA-I−/−, apoE−/− double knockout mice have identified apoE as a potential LCAT activator [37]. In an in vitro model of reconstituted HDL that lacks apoA-I, apoE activates LCAT [38]. In contrast, apoC-III dose-dependently inhibits the LCAT reaction in reconstituted HDL [33], and in synthetic phosphatidylcholine vesicles containing apoA-I [39]. Therefore, the finding of no decrease in LCAT activity in apoC-III-containing HDL was unexpected.

Contrary to LCAT, the concentration and activity of CETP did not vary among HDL subtypes. Some CETP inhibitors increase both plasma apoC-III and plasma apoE [40–42], but findings from this study suggest that CETP activity and concentration in HDL are not influenced by these apolipoproteins. CETP activity in humans is known to be influenced by high plasma cholesterol concentrations, or by cholestasis [43, 44].

Concerning the lipid composition of HDL subtypes, there was more phosphatidylcholine in HDL with either apoE or apoC-III. Thus, higher LCAT activity in these HDL subtypes may be a consequence of greater substrate availability. This is a plausible explanation, as the movement of apolipoproteins between lipoproteins sometimes involves phospholipid transfer protein (PLTP), in which cases there may be simultaneous transfer of the surrounding phospholipid-rich membrane [45]. There was no statistically significant difference in the cholesteryl ester content of the four HDL subtypes.

Total plasma LCAT activity showed a significant inverse correlation with the concentration of both E-C+ pre-beta HDL and E-C- alpha 1 HDL. These results suggests that the presence of apoC-III in pre-beta HDL may correlate with limited LCAT activity, less maturation and accumulation of pre-beta HDL [46]. Interestingly, a study comparing patients with and without CVD found an inverse correlation between LCAT activity and plasma pre-beta HDL concentrations that was stronger in the CVD group [47]. The negative correlation between E-C- alpha 1 HDL and plasma LCAT activity suggests that not all size conversion/maturation of HDL requires LCAT. Meanwhile, the positive correlation of plasma CETP activity with the abundance of large E-C+ HDL may just reflect the frequent coexistence of both proteins (CETP and apoC-III) in this HDL type.

Strengths of this study include the exploration of central aspects of HDL functionality in the real physiological context of living humans. ApoE and apoC-III are biochemical antagonists [48] with an opposite impact on HDL metabolism and cardiovascular risk [49], making them particularly worthy of investigation. The finding of an association between apoE in HDL and cholesterol esterification has clinical relevance, because the influence of apoE on HDL metabolism is susceptible of positive modification through dietary intervention [50].

The main limitations of the study are its limited sample size and the fact that only two specific apolipoproteins were studied, out of the many known to be present in HDL. However, this was a proof-of-concept study in which the core analyses were performed within-individual, so that enzymatic contents and activities were performed for different HDL subtypes belonging to the same participant. Another possible limitation is that the employed LCAT activity assay probably detects only the fatty acid removal from phosphatidylcholine and not its successful transfer to free cholesterol. Nonetheless, given the known nature of the LCAT enzymatic process, the ability of LCAT to perform this initial cleavage in vitro has been widely employed as a proxy for LCAT activity.

## Conclusion

The results of this study showed specific profiles of LCAT mass and activity in HDL subtypes defined by their content of apoE and apoC-III. The presence of apoE protein in HDL was a correlate of increased LCAT activity, suggesting that LCAT and apoE interact to enhance anti-atherogenic pathways dependent on HDL functionality. Further studies describing the changes in HDL composition, functionality and associated enzymatic activity in the context of CVD would expand on these findings.

## Data Availability

The datasets used and/or analysed during the current study are available from the corresponding author on reasonable request.

## Abbreviations

apoC-III: apolipoprotein C-III
apoE: apolipoproteins E
AU: Arbitrary Units
BMI: Body-mass index
CETP: Cholesterol-ester transfer protein
CVD: Cardiovascular disease
HDL: High density lipoproteins
HRP: Horseradish peroxidase
LCAT: Lecithin-cholesterol acyl transferase
LRP-1: LDL receptor-related protein-1
RCT: Reverse cholesterol transport
PUFA: Polyunsaturated fatty acids
PVDF: Polyvinylidene fluoride
